# Severe cerebral involvement due to idiopathic systemic capillary leak syndrome

**DOI:** 10.1002/ccr3.525

**Published:** 2016-03-16

**Authors:** Ali Riza Günes, Peter Berlit, Ralph Weber

**Affiliations:** ^1^ Department of NeurologyAlfried‐Krupp‐Hospital EssenAlfried‐Krupp‐Str. 2145131EssenGermany

**Keywords:** coma, critical care, neurology, systemic disease

## Abstract

The idiopathic systemic capillary leak syndrome (ISCLS) is a rare disorder, characterized by recurrent attacks of hypotension, hypoalbuminemia, and hemoconcentration, which is often misdiagnosed due to overlapping features with other diseases. Even though cerebral involvement is uncommon, a broad awareness is crucial, because of its life‐threatening character.

## Case

We report the case of a 43‐year‐old woman who was referred to our hospital with coma of unknown origin. Ten days earlier, she developed gastro‐enteritic complaints with nausea and vomiting, followed by confusion and expressive aphasia, leading to admission to an internal medicine service. Physical examination revealed a slightly elevated temperature of 37.9°C, normal blood pressure and oxygen saturation, and slight peripheral edema. Laboratory findings showed hypoalbuminemia (2.2 g/dL) in the absence of albuminuria, hemoconcentration (hematocrit 55%), and a known IgG monoclonal gammopathy.

**Figure 1:**
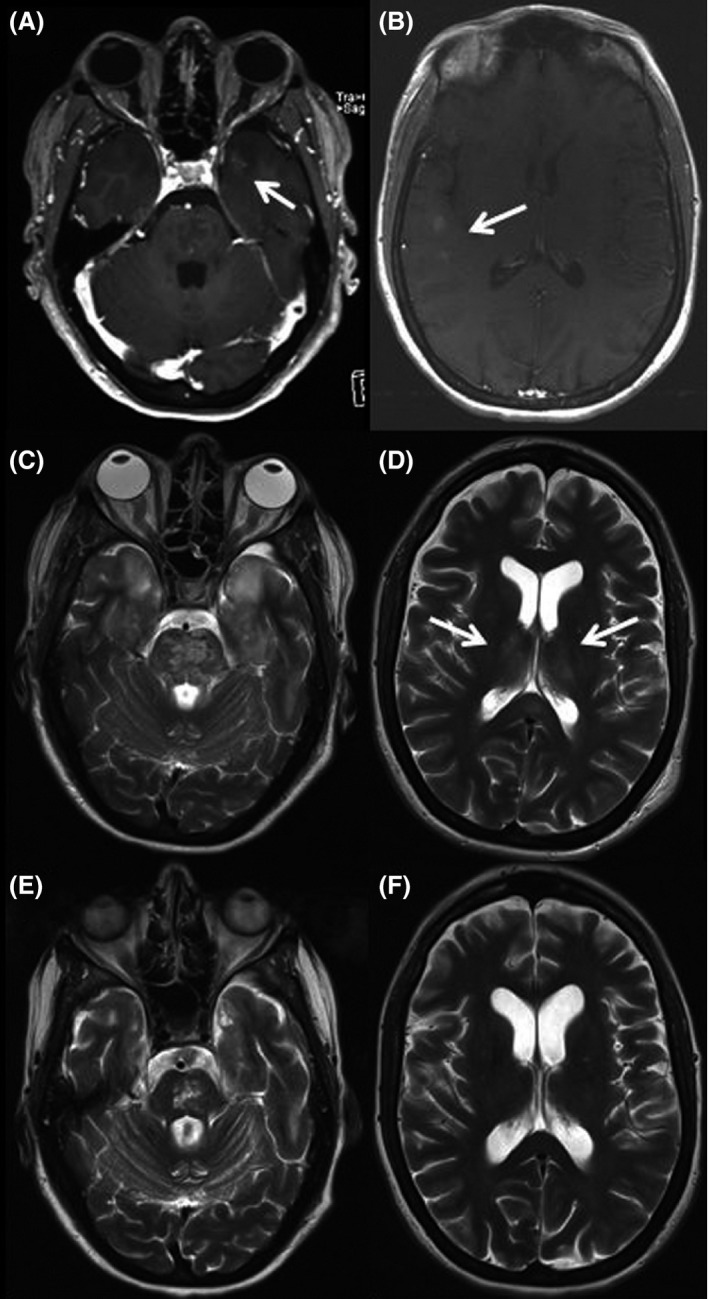
Serial contrast‐enhanced (A + B) and T2‐weighted (C‐F) MRI.

The initial MR of the brain showed contrast agent uptake in the temporal lobe of both hemispheres (Fig. 1A and B) and the posterior part of the corpus callosum.

All inflammatory parameters (including ANA, ENA, ANCA antibodies, and cerebrospinal fluid (CSF) findings) were normal, therefore a (cerebral) vasculitis as a differential diagnosis was unlikely. Osmotic demyelination syndrome was also discarded since there were no observable electrolyte abnormalities during the clinical course. Assays for neurotropic viruses, paraneoplastic antibodies and HIV were also negative.

The patient's clinical condition worsened with generalized edema and coma requiring mechanical ventilation. One week after symptom onset, she was transferred to our intensive care unit and MRI showed massive, symmetrical T2‐weighted lesions with concomitant diffusion restriction in the brain stem (Fig. 1C), cerebellum, hippocampi, thalami (Fig. 1D), and posterior part of the corpus callosum.

The brother of the patient reported of two previous attacks with confusion, hypotension, and generalized edema following respiratory infections. The last attack in 2011 also resulted in progressive vigilance deterioration with coma and mechanical ventilation. T2‐weighted MRI at this time showed symmetrical hyperintensities in both thalami. The patient completely recovered from this attack and a diagnosis of cerebral venous thrombosis was made despite missing evidence of sinus thrombosis in venous MR angiography. Follow‐up MRI was normal. No other family member was affected so far.

A diagnosis of idiopathic systemic capillary leak syndrome (ISCLS) was made because of recurrent attacks of generalized edema, hypotension, hypoalbuminemia, and hemoconcentration and a concomitant monoclonal gammopathy, which has been reported in the vast majority of patients 1, 2.

We administered high‐dose (2 g/kg body weight) intravenous immunoglobulin (IVIG) and were able to extubate the awake patient 2 weeks later. Follow‐up MRI showed regressive T2‐weighted lesions in the brainstem, both temporal lobes and thalami (Fig. 1E and F). Over the next 3 months, the patient partially recovered and was able to speak slowly and to swallow, but a high‐grade right hemiparesis persisted. Prophylactic monthly therapy with IVIG was initiated.

## Discussion

Idiopathic systemic capillary leak syndrome (ISCLS) is a rare and life‐threatening disorder first described by Clarkson and is characterized by recurrent attacks of hypotension, hypoalbuminemia, and hemoconcentration 1, 2, 3. The cause of ISCLS is unknown. Pathophysiologically, a profound dysfunction of the vascular endothelium leading to leakage of plasma and proteins from the blood vessels into extravascular spaces is supposed. To maintain hemodynamic stability and prevent organ failure due to hypoperfusion, conservative fluid replacement is essential during the leak phase of fluid mobilization. Nevertheless, a certain amount of fluid therapy raises the risk of acute complications such as acute pulmonary edema and compartment syndrome 4.

Another disease, where hemoconcetration may occur is polycythemia vera (PCV) 5. However, hypoalbuminemia, hypotension and recurrent neurological deficits with cerebral MRI changes are not common features in PCV. Disseminated T2‐weighted cerebral hyperintensities are consistent with the radiomorphological diagnosis of an acute demyelinating disease like Marburg's variant of Multiple sclerosis 6, progressive multifocal leucoencephalopathy, or CNS lymphoma. Normal CSF findings including the absence of JC Virus DNA and recurrent attacks with spontaneous recovery in the history of our immunocompetent patient made these diagnoses unlikely. Repetitive episodes of shock without symptoms of a capillary leak have been described in toxic shock syndrome in women, mast cell disease, and adrenal insufficiency 7, 8, 9.

A massive and multilocular involvement of the blood–brain barrier in ISCLS as shown in our case has not been described in adults before. There is one study describing a cerebral involvement in a child with ISCLS 10. To the best of our knowledge, only one other case with cerebral involvement leading to unilateral malignant cerebral infarction in adults has been published to date 11. IVIG seems to be a rational treatment option for the acute phase in patients with ISCLS and cerebral involvement 12. Theophylline and Terbutaline have also been reported to reduce the frequency of attacks 13.

## Conflict of Interest

The study was not sponsored. Written consent for publication was obtained from the patient and husband.
